# Strong Coupling between Surface Plasmon Resonance and Exciton of Labeled Protein–Dye Complex for Immunosensing Applications

**DOI:** 10.3390/ijms24032029

**Published:** 2023-01-19

**Authors:** Povilas Jurkšaitis, Ernesta Bužavaitė-Vertelienė, Zigmas Balevičius

**Affiliations:** Plasmonics and Nanophotonics Laboratory, Department of Laser Technologies, Center for Physical Sciences and Technology, Sauletekio Ave. 3, LT-10257 Vilnius, Lithuania

**Keywords:** Rabi gap, surface plasmon resonance, strong coupling, human serum albumin, AlexaFlour^TM^ 633

## Abstract

In this study, we present an analysis of the optical response of strong coupling between SPR and labeled proteins. We demonstrate a sensing methodology that allows to evaluate the protein mass adsorbed to the gold’s surface from the Rabi gap, which is a direct consequence of the strong light–matter interaction between surface plasmon polariton and dye exciton of labeled protein. The total internal reflection ellipsometry optical configuration was used for simulation of the optical response for adsorption of HSA-Alexa633 dye-labeled protein to a thin gold layer onto the glass prism. It was shown that Rabi oscillations had parabolic dependence on the number of labeled proteins attached to the sensor surface; however, for photonic–plasmonic systems in real experimental conditions, the range of the Rabi energy is rather narrow, thus it can be linearly approximated. This approach based on the strong coupling effect paves the alternative way for detection and monitoring of the interaction of the proteins on the transducer surface through the change of coupling strengths between plasmonic resonance and the protein–dye complex.

## 1. Introduction

Immunosensing based on biomarkers has emerged as a tool for various disease research, such as cancer [1], cardiovascular diseases [2,3], and other health-related issues [4]. Particular research has been widely employed for proteins labeled with fluorescent biomarkers used in biosensing due to their low detection limit [4,5,6] and wide range of fluorescent dye molecules suitable for biomarkers to select from. This allows using biomarkers as a cheap and versatile tool for immunosensing. However, the fluorescent dyes can experience photobleaching due to the high probability of the long-lived triplet states of the fluorophore and environmental oxygen [7] interacting. This results in depletion of the dye’s fluorescence lifetime and, as a result, reduces the signal intensity. At low concentrations, due to the contribution of photobleaching, the measured intensity decreases further and the detection of the analyte can be complicated. Since fluorescence-based immunosensing methods are sensitive, cost-effective, and time-saving, various signal enhancement techniques have been used [8,9,10,11].

One of the most recent approaches of photobleaching suppression is the application of metal nanostructures, which can enhance the optical properties of the fluorophores and improve the output fluorescence intensity and quantum yield [12,13], thus, the detection limit of proteins can increase. The augmentation of the fluorophore’s optical properties is due to the electric field localization when a plasmonic excitation is generated in the metallic nanostructure. However, even though fluorescent dye-labeled methods have a low detection limit of ~tens pm/mL [6], the broader application of these methods is limited due to the photobleaching effect, as the long photobleaching times (up to tens of minutes) can distort the real-time immunosensing kinetics. For this reason, a label-free measuring technique based on surface plasmon resonance (SPR) has become popular [14]. Surface plasmon polaritons are propagated surface electromagnetic waves excited at the metal/dielectric boundary. Such light–matter interaction allows the squeezing of light into subwavelength volumes. The SPR is generated at a specific frequency due to the interaction between the incident electromagnetic field and collective oscillation of conduction electrons at the metal’s surface. In order to generate the SPR, the wave vector matching conditions must be met, as the plasmons’ in-plane wave vector is larger than that of light in vacuum. For this reason, light couplers such as a prism or grating are used to excite the SPR. The SPR has been extensively used in biosensors as a tool to measure protein kinetics [15] but also adsorbed protein mass that is usually determined by Feijter’s formula [16]. The emergence of SPR came due to the high spectral sensitivity of the mode, however, the enhancement of the SPR sensitivity is limited by the absorption and scattering losses [17,18] in the metal layer. As a result, a solution to reduce the losses and enhance the sensitivity of the SPR is needed [19,20,21]. By combining the plasmonic resonance and excitons of fluorescent dye-labeled proteins, the disadvantages of both methods, SPR (losses in the metal) and fluorescent biosensing (photobleaching), can be minimized. If the right conditions for the plasmon and exciton to be able to interact with each other are arranged, an enhancement of the fluorescence can occur due to the coupling of the modes. Depending on the geometry of the structures, the distance between excitations, and their energies, the coupling can be either weak or strong. In the weak coupling regime, the energy dissipates faster than coherent energy exchange between the modes happens. On the contrary, when the coupling exceeds the damping, a strong coupling regime is reached and the excitations of the hybrid plasmon–exciton mode exchange energy coherently (coherent Rabi oscillations) at a rate of tens of femtoseconds [22]. This strong light–matter interaction can allow modifying not only electromagnetic [23] but material properties as well, such as controlling chemical reactions [24,25,26]. The possibility to control chemical reactivity by means of strong coupling could open up new possibilities to alter photobleaching in immunosensing applications. The effect of strong coupling between plasmons and excitons is extensively researched in metallic nanostructure and J-aggregate systems [27], while a small amount of research has been conducted on photochemical reactions and biosensing applications. Recently, it has been theoretically shown that in quantum plasmonic immunoassays, the sensitivity of the sensor can be enhanced by strong coupling between plasmons of metal nanoparticles and the fluorescent dye-labeled protein exciton [28]. It was also demonstrated that the photo-oxidation of the organic molecules can be suppressed by means of strong coupling [29]. However, the strong coupling between surface plasmons and excitons of fluorescent dye-labeled protein and the Rabi gap’s relation to the adsorbed protein mass has been studied.

In this research, we present a theoretical study of strong coupling between SPR and labeled protein. We propose and demonstrate new sensing methodology that allows to evaluate the adsorbed protein mass to the metal surface from the Rabi gap of strongly coupled components in the hybrid polaritonic modes, rather than from refractive index changes used in label-free biosensing methods. Since the Rabi gap is proportional to the ratio between number of particles participating in strong coupling and the volume, it is a direct method to evaluate the number of excitons participating in strong coupling. If the labeled proteins are immobilized to the sensors’ surface, the number of attached protein molecules will be proportional to the number of excitons participating in strong coupling.

## 2. Results and Discussion

**Figure 1:**
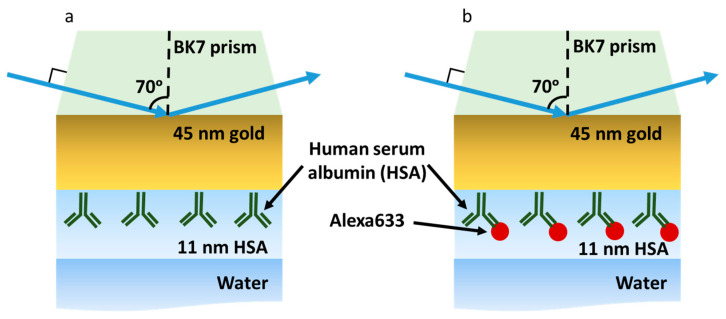
The schematic representation of the two modeled structures: (**a**) BK7/Au (45 nm)/HSA and water and (**b**) BK7/Au (45 nm)/HSA-Alexa633 and water.

The labeled and non-labeled proteins are widely used for various immunosensor applications. In this study, a model consisting of thin gold (45 nm) and a protein layer sandwiched between BK7 glass on one side and water on the other (Figure 1) was used. The p-polarized reflectance of the two multilayer structures involving human serum albumin (HSA) proteins (Figure 1a) and HSA labeled with Alexa Fluor^TM^ 633 dye (further Alexa633 will be used) complex (Figure 1b) have been compared and investigated. The optical response of two multilayer structures, water/HSA (11 nm)/Au (45 nm)/BK7 and water/Alexa633-HSA (11 nm)/Au (45 nm)/BK7, was simulated in total internal reflection (TIR) ellipsometric configuration. It should be noted that the surface plasmon resonance phenomenon is commonly used in commercial optical label-free biosensors because the sensing surface is sensitive to refractive index changes [30] of the ambient environment, thus the SPR dip shifts to a longer wavelength or larger angle of incidence (AOI) of the p-polarized light, what is finally proportional to the adsorbed protein mass on the sensor’s surface.

**Figure 2:**
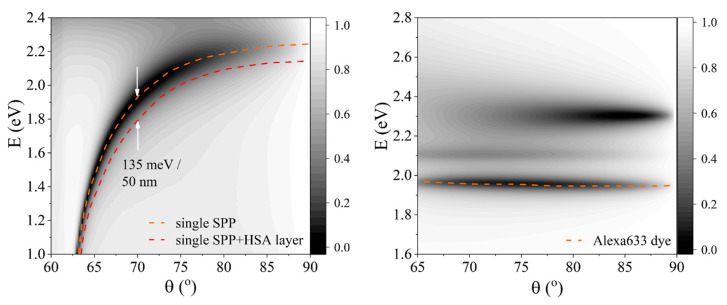
The p-polarization reflected intensity maps of the SPP (**left**) and exciton (**right**) dispersion relations. The orange lines mark the SPP excitation minima and the Alexa Fluor^TM^ 633 dye absorption peak, while red line represents the minima of SPP when 11 nm HSA protein layer is formed on gold’s surface, where white arrows mark the 50 nm spectral shift (at 70° AOI) due to the presence of 11 nm HSA layer.

**Figure 3:**
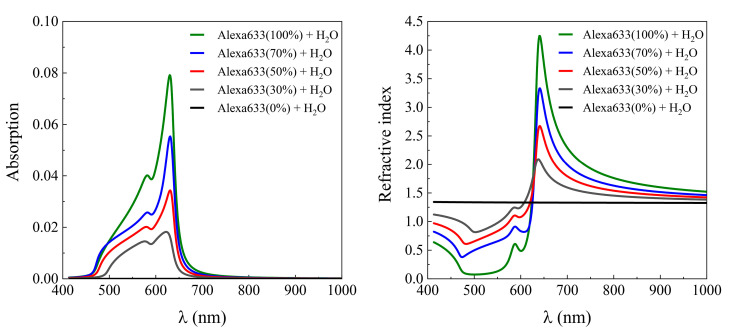
Absorption (**left**) coefficients and refractive index (**right**) of the HSA protein labeled with Alexa Fluor^TM^ 633 dye.

Firstly, the optical response of the label-free HSA protein layer was investigated (water/HSA/Au/BK7). For the excitation of surface plasmon mode in metal layers, the thickness of the metal must be of the order of 10’s of nanometers; therefore, for our purposes, a thickness of 45 nm for the gold layer was chosen. The shift of plasmonic resonance to the longer wavelength due to the presence of the HSA protein layer was 50 nm (Figure 2) at 70° AOI. The refractive index of protein has been modeled using the Cauchy function for normal dispersion of the HSA protein molecule in the visible range with coefficients A = 1.567, B = 0.0022, and C = 0. The typical thickness of the HSA molecule has been observed to be dependent on the orientation of the structure [31], however, the average value can be estimated to be around 11 nm. In this modeling, we assume that HSA proteins bind to the gold surface randomly. Simulations have been performed with a wavelength range from 400 nm to 1000 nm of irradiated light for angles of incidence from 60° to 90°. The typical SPR-based optical biosensor is a label-free method, thus, the shift of plasmonic resonance due to the binding of non-labeled HSA was used as a reference signal indicating an adsorbed surface mass of protein molecules. Secondly, the non-labeled HSA protein layer was replaced by the dye-labeled protein HSA-Alexa633 complex. The dye-labeled protein layer was modeled as an HSA-Alexa Fluor^TM^ 633 dye complex, where HSA refractive index dispersion was described by the Cauchy function while the fluorescent dye absorption lines were modeled as two Lorentz oscillators. The energies of the oscillator peaks were at 1.96 eV and 2.13 eV and the modeled absorption spectra (Figure 3) were taken from the experimentally measured Alexa Fluor^TM^ 633 dye complex [32].

The SPR resonant wavelength dependence on AOI dispersion maps when a non-labeled HSA layer was formed is presented in Figure 3a. As mentioned earlier, the SPR shifts to longer wavelengths when a protein layer is attached to the gold’s surface. On the contrary, the fluorescent dye Alexa Fluor^TM^ 633 is non-dispersive, thus it does not depend on AOI (Figure 3b). However, when the structure water/HSA-Alexa633/Au/BK7 is arranged in a way that the optical dispersion of the SPR and Alexa Fluor^TM^ 633 dye exciton cross, a new polaritonic state is created. If the electric fields of the exciton and plasmon overlap significantly, the spectral changes become experimentally detectable when the shifts of the modes are larger than the width of the resonances. It should be noted that in the range where the shift of the two coupled modes exceeds the linewidth, the system is in the strong coupling regime [33]. The energy gap between the two shifted excitations at the SPR and exciton dispersions’ crossing point is called Rabi splitting. As a result, the new polaritonic states become inextricably linked, which in turn modifies the reflectance spectra and can be directly measured by spectroscopic ellipsometry.

**Figure 4:**
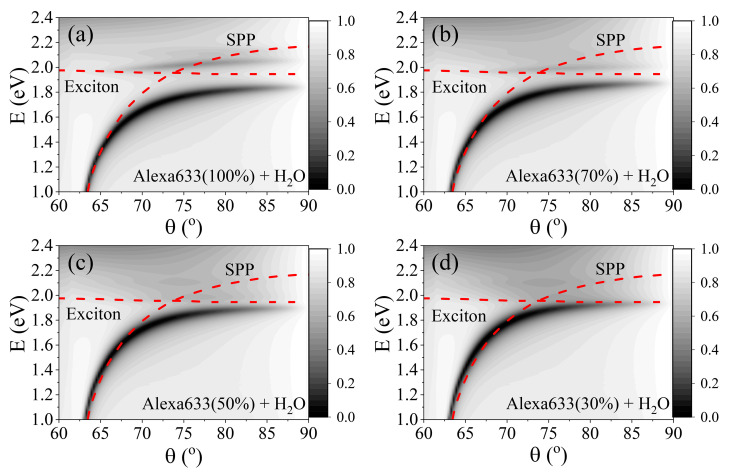
Dispersion maps of hybrid SPP–exciton modes for different Alexa633/H20 mixture ratios: (**a**) 100% Alexa Fluor^TM^ 633, (**b**) 70%, (**c**) 50% and (**d**) 30%.

As can be seen from Figure 4, a new hybrid mode between the SPR and Alexa Fluor^TM^ 633 dye-labeled protein layer was created. In order to study the strong coupling dependence on the fluorescent dye concentration, a water/Alexa633-HSA (11 nm)/Au (45 nm)/BK7 structure was investigated. The dye-labeled HSA-Alexa633 protein complex was modeled as a thin layer of Alexa633 dye and HSA protein complex and deionized water with different mixing ratios, indicated as percentage of protein dye contained in the layer, ranging from pure protein dye (100%) to deionizing water (0%).

The mixture of optical dispersions of the two materials, the deionized water, and the HSA-Alexa633 complex produce different refractive indices and extinction coefficients for different concentrations, thus the mixing ratio must be taken into account. The optical response of the ellipsometric parameters of effective dielectric functions has been modeled using effective media approximation (EMA) Bruggeman’s model. Simulated dispersion curves of EMA HSA-Alexa633/water layers with different concentrations are shown in Figure 3, where the main absorption peak of the Alexa633 dye can be seen at 632 nm and another at 583 nm. The absorption and refractive index spectra of 100% concentration Alexa633 protein dye are shown in Figure 3 as a green line. With a decreasing number of HSA-Alexa633 complex molecules, the absorption and refractive index coefficients gradually approach the dispersion relation of the deionized water.

A single molecular exciton dispersion line can be seen in Figure 2 (right). The exciton quasiparticle induced in protein dye Alexa633 of 100% concentration was modeled as a function of the angle of incidence and shows no dependence on the angle. Several dispersion lines appear in Figure 2 that correspond to different peaks of molecular absorption, with maximum absorption around 1.96 eV (orange dashed line). A single dispersion line of uncoupled SPP shows a dispersion relation on the angle of incidence.

By varying the angle of incident light on the sample, the p-polarized light of the wave vector k changes the magnitude at the boundary between the prism coupler and gold nanolayer. Since the excitation of SPP is dependent on the wave vector magnitude, the dispersion relation can be found. Simulations of SPP and exciton dispersions were performed in order to evaluate the dependence of coupling strength as a function of labeled protein concentration. Numerical simulations for different protein dye concentrations between 100% and 0% were performed. The introduction of labeled protein molecular excitons on top of the 45 nm thick gold film forms a hybrid SPP and molecular excitons mode. The dispersion lines of the strongly coupled modes at different Alexa633 concentrations are shown in Figure 4, where the red dashed lines indicate the dispersion relations of a single SPP mode and single exciton.

The numerical simulation has shown that the SPP and exciton are modified compared to their initial optical dispersions and lower and upper polariton branches of new eigenmode are formed (SPP, light-like, and Alexa633 dye exciton, matter-like), indicating that the system is in a strong coupling regime. As can be seen from Figure 4a, when the concentration of Alexa633 is 100%, the gap between the new hybrid mode at the anti-crossing of single modes point (AOI = 74°) is equal to 264 meV at 74° AOI. The energy gap between the modes decreases with the decreasing concentration of HSA-Alexa633 molecules (Figure 4a–d), as a result, the Rabi splitting between the strongly coupled matter-light components decreases. The strength of interaction between the SPP and molecular excitons at 74° AOI decreased to 198 meV and 161 meV for the 70% and 50% concentrations, respectively, while for concentrations less than 30%, the strong coupling almost vanishes due to a smaller number of excitons participating in the strong coupling. The Rabi frequency (Ω) of strongly coupled modes can be derived by Equation (1) [33], where the Ω is directly proportional to the number of particles (N/V) that participate in the strong coupling:

(1) $$\Omega =\frac{e}{\sqrt{\varepsilon_{0}m}}\sqrt{\frac{N}{V}}$$

where the *ε*_0_ is the vacuum dielectric constant (8.85 × 10^−12^ Fm^−1^), *e* is the charge of the electron (1.602 × 10^−19^ C), and *m* is the electron’s mass (9.109 × 10^−31^ kg). Thus, the measurement of the Rabi gap could be a direct tool for evaluating the number of proteins adsorbed to the gold surface:

(2) $$\frac{N}{V}=\frac{\Omega^{2}\varepsilon_{0}m_{e}}{e^{2}}$$

**Figure 5:**
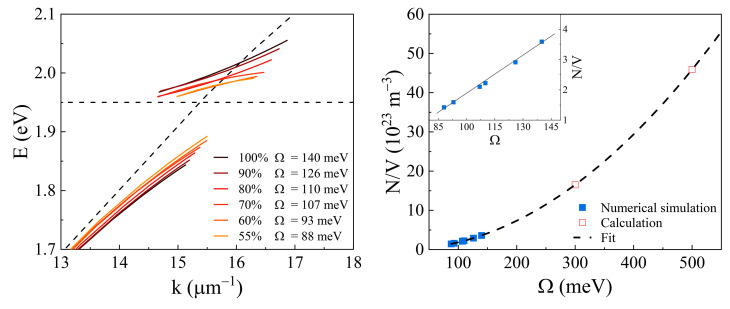
Calculations of SPP–exciton wave vector dispersion in the strong coupling regime for different concentrations (55–100%) of protein–dye complex (**left**) and adsorbed protein molecule concentration dependence on modeled Rabi energy gap (**right**). The black dashed lines on the left indicate the dispersions of single SPP and exciton. The blue squares on the right indicate the numerically simulated, while the red squares are calculated Ω values.

To analyze the Rabi energy gap between polaritonic states, the dispersion relation as a function of the in-plane wave vector was calculated (Figure 5a). Evaluations have shown the dependence of the coupling strength as a function of dye concentration. The Rabi energy gap values vary between 140 meV and 88 meV, where the gap increases with a higher concentration of labeled protein (from 55% to 100%). Furthermore, the coupling strength could be increased only by introducing a higher number of particles participating in the coupling (pure fluorescent molecules without protein).

The number of proteins adsorbed to the surface can be evaluated from the Rabi gap by using Equation (2). Using the results from wave vector dispersions (Figure 5 left), the number of adsorbed molecules of the HSA-Alexa633 complex was calculated. To simulate a full gold surface coverage, the number of HSA-Alexa633 complex molecules in volume (N_total_) has to be higher than the number of molecules bound to the gold fully covering the surface (N). Since the electromagnetic field of the SPP mode is confined at the gold/water boundary, the SPP and exciton can interact strongly at a small distance (tens of nm) if the electric fields of these modes significantly overlap. The thickness of the adsorbed dye-labeled protein layer is similar to the distance between the SPP and excitons that participate in the strong coupling. As a result, the number of labeled molecules is proportional to the adsorbed protein mass. Since the thickness of the modeled HSA-Alexa633/water layer is 11 nm thick, the number of molecules in volume (N/V) is proportional to the number of particles adsorbed to the surface. To simulate the full coverage case, the fill factor (N_vol_/N) must be equal to 1, thus, the fill factor of the HSA-Alexa633 complex was chosen to be 100%. The Rabi energy gap was Ω = 140 meV (3.385 × 1013 s^−1^), and thus, based on Equation (2), the number of molecules adsorbed to the gold surface (density of states) was N/V = 3.595 × 10^23^ m^−3^.

As the number of molecules (N/V) is proportional to Ω^2^, the concentration of the HSA-Alexa633 complex dependence on the Rabi gap in the water/HSA-Alexa633/Au/BK7 structure was evaluated (Figure 5 right). The calculated values (red square) have a parabolic relation (fitted curve). However, since the Rabi gap in simulated plasmon-based nanophotonic structures varies in a rather narrow range (Figure 5 (right) blue squares), the changes in Ω on concentration can be approximated by linear curve and the dependence can be considered to be N/V ~ Ω (Figure 5 right inset). This modeling of optical response demonstrates the influence of the strong coupling on the emitting dye molecules on the sensor surface, thus the changes in the vacuum Rabi gap could be a useful indication for changes in the number of attached HSA–dye protein complexes.

## 3. Materials and Methods

The optical response of two multilayer structures, water/HSA (11 nm)/Au (45 nm)/BK7 and water/Alexa633-HSA (11 nm)/Au (45 nm)/BK7, was simulated using CompleteEase software in total internal reflection (TIR) ellipsometric configuration. The optical properties of BK7, gold and water materials were taken from CompleteEase database, while the HSA was modeled as Cauchy function (with coefficients A = 1.567, B = 0.0022, and C = 0) and the Alexa Fluor^TM^ 633 dye absorption properties were taken from Thermo Fisher Scientific database [32].

## 4. Conclusions

Labeled as well as label-free proteins are widely used for various immunological methods to monitor the dynamics of antibodies and antigens and predict the interaction of proteins. The SPR-based label-free method is one of the commonly used optical methodologies for immunosensing applications. This study presents the combination of a label-free optical method with labeled proteins by employing the strong coupling effect between plasmonic resonance and dye exciton of target proteins. This new approach paves the alternative way for the detection and monitoring of the protein’s interaction on the transducer surface through the change of coupling strengths between plasmonic resonance and emitter. It was shown that Rabi oscillations had parabolic dependence on the number of labeled proteins attached to the sensor surface; however, for photonic–plasmonic systems in real experimental conditions, the range of the Rabi energy is rather narrow, thus it can be linearly approximated. Employing the strong light–matter coupling for immuno-complexes should allow changing chemical reactivity, excited states relaxation pathways, charge conductivity, and rates of binding kinetics of proteins. Moreover, such bio-polaritonic states, which involve proteins, can be used for quantum information processing. The development of strongly coupled states between plasmonic resonances and protein–dye complexes promise new concepts of biosensing where the combination of label-free and labeled methods gives the possibility to tune binding kinetics rates of proteins’ interactions through the coupling strength, the bio-nanolasers concept, and the pathways for polaritonic biochemistry.

## Data Availability

Not applicable.
